# C2-Phytoceramide Perturbs Lipid Rafts and Cell Integrity in *Saccharomyces cerevisiae* in a Sterol-Dependent Manner

**DOI:** 10.1371/journal.pone.0074240

**Published:** 2013-09-11

**Authors:** Andreia Pacheco, Flávio Azevedo, António Rego, Júlia Santos, Susana R. Chaves, Manuela Côrte-Real, Maria João Sousa

**Affiliations:** 1 CBMA (Centre of Molecular and Environmental Biology), Department of Biology, University of Minho, Braga, Portugal; 2 Life and Health Sciences Research Institute (ICVS), School of Health Sciences, University of Minho, Braga, Portugal; 3 ICVS/3 B’s - PT Government Associate Laboratory, Braga/Guimarães, Portugal; Florida State University, United States of America

## Abstract

Specific ceramides are key regulators of cell fate, and extensive studies aimed to develop therapies based on ceramide-induced cell death. However, the mechanisms regulating ceramide cytotoxicity are not yet fully elucidated. Since ceramides also regulate growth and stress responses in yeast, we studied how different exogenous ceramides affect yeast cells. C2-phytoceramide, a soluble form of phytoceramides, the yeast counterparts of mammalian ceramides, greatly reduced clonogenic survival, particularly in the G2/M phase, but did not induce autophagy nor increase apoptotic markers. Rather, the loss of clonogenic survival was associated with PI positive staining, disorganization of lipid rafts and cell wall weakening. Sensitivity to C2-phytoceramide was exacerbated in mutants lacking Hog1p, the MAP kinase homolog of human p38 kinase. Decreasing sterol membrane content reduced sensitivity to C2-phytoceramide, suggesting sterols are the targets of this compound. This study identified a new function of C2-phytoceramide through disorganization of lipid rafts and induction of a necrotic cell death under hypo-osmotic conditions. Since lipid rafts are important in mammalian cell signaling and adhesion, our findings further support pursuing the exploitation of yeast to understand the basis of synthetic ceramides’ cytotoxicity to provide novel strategies for therapeutic intervention in cancer and other diseases.

## Introduction

Ceramide has emerged as an important second-messenger lipid with proposed roles in a wide range of cellular processes such as cell growth, differentiation, apoptosis, stress responses, and senescence. Ceramide can activate enzymes involved in signaling cascades comprising both protein kinases and phosphatases, such as ceramide-activated protein kinase (CAPK) and ceramide-activated protein phosphatases (CAPPs) [1]. CAPK regulates several kinases, including the mitogen activated protein kinase (MAPK) ERK (extracellular-signal regulated kinase), leading to cell cycle arrest and cell death, stress-activated protein kinases (SAPKs) such as the Jun kinases (JNKs) and p38-MAPK, kinase suppressor of Ras (KSR), and the atypical protein kinase C (PKC) isoform zeta [2,3]. Ceramide activation of CAPPs, which comprise the serine threonine protein phosphatases PP1 and PP2A [1,4], leads to dephosphorylation and inactivation of several substrates, such as Bcl-2 and Akt [1], and downregulation of the transcription factors c-Myc and c-Jun [3,4]. Ceramide and sphingosine levels increase in response to stress and in apoptosis induced by several stimuli such as FAS activation and anticancer drugs, and ceramides regulate mammalian apoptosis by both transcriptional-dependent and -independent mechanisms [3]. Receptor clustering and apoptosis induced by death ligands, such as FAS and TNF alpha, involves ceramide generation by sphingomyelinase acting primary in lipid rafts [2].

The yeast *Saccharomyces cerevisiae* has been extensively used in the elucidation of numerous cellular and molecular processes that have proven conserved across species, such as cell cycle control and apoptosis [5]. Several studies indicate that the ceramide pathway is a ubiquitous signaling system, conserved from yeast to human [6]. Exogenous N-acetylsphingosine (C2-ceramide) specifically inhibited proliferation of *S. cerevisiae*, inducing an arrest at G1 phase of the cell cycle mediated by yeast CAPP [7]. Similarly to mammalian cells, yeast ceramide levels increase in response to stress [8], and perturbations in sphingolipid metabolism can also determine yeast cell fate. Expression of mammalian sphingomyelin synthase (SMS1) suppresses Bax-mediated yeast cell death and confers resistance to different apoptotic inducers [9], suggesting that SMS1, which uses ceramide to synthesize sphingomyelin, protects cells against death by counteracting stress-induced accumulation of the pro-apoptotic ceramide. Yet another study showed that a yeast mutant deficient in Isc1p, a member of the neutral sphingomyelinase family, displays increased apoptotic cell death in response to hydrogen peroxide and during chronological aging [10]. A lipidomic approach revealed that these phenotypes were associated with increased levels of dihydro-C26-ceramide and phyto-C26-ceramide [11]. Very recently it has also been reported that some phytoceramides contribute to cell death induced by acetic acid, and are involved in mitochondrial outer membrane permeabilization [12]. Another study showed that exogenous C2-ceramide can trigger a mitochondria-mediated cell death process in yeast [13].

In summary, literature data indicate that exogenous ceramides and changes in the levels of endogenous ceramides, as well as other sphingolipids such as sphingosine, dihydroceramide and phytoceramide, can affect cell fate in yeast [8]. Since yeast and mammals share many similarities in sphingolipid metabolism [14], we aimed to explore *S. cerevisiae* as a model system to advance our knowledge on the molecular basis of ceramide-induced cell changes, as well as of the involvement of signaling pathways in this process. We show that exogenous C2-phytoceramide (N-acetyl-D-phytosphyngosine) induces growth arrest in the G0/G1 phases and loss of clonogenic survival in the G2/M phases. Defects in cell wall and plasma membrane integrity, resulting in higher sensitivity to osmotic stress, seem to underlie loss of survival. C2-phytoceramide disturbed lipid rafts and caused higher intracellular accumulation of sterols, suggesting the observed phenotypes are a result of defects in trafficking. We also show that C2-phytoceramide-treated cells require the HOG (High Osmolarity Glycerol) pathway for the response against cytotoxicity induced by C2-phytoceramide, but not the cell wall integrity pathway.

## Materials and Methods

### Yeast Strains

The yeast *S. cerevisiae* strain W303-1A (*MATa*, *ura3-52*, *trp1Δ 2, leu2-3,112*, *his3-11*, *ade2-1*, *can1-100*) was used throughout this work as the wild type strain. *S. cerevisiae* strain BY4741 was also used to test sensitivity to C2-phytoceramide. All the mutant strains were constructed by replacing the respective genes in the W303-1A strain with a *kanMX4* disruption cassette, amplified by PCR from genomic DNA purified from the respective Euroscarf deletion strain as described in the *Saccharomyces* Genome Deletion Project database [15].

### Media and growth conditions

Cells were maintained on YPD agar plates containing glucose (2%), yeast extract (1%), peptone (2%) and agar (2%) and grown in liquid synthetic complete medium (SC) [(0.67% Yeast nitrogen base without amino acids, galactose (2%), 0.14% drop-out mixture lacking histidine, leucine, tryptophan and uracil, 0.008% histidine, 0.04% leucine, 0.008% tryptophan and 0.008% uracil] until mid-exponential phase.

### Cell Viability Assays

W303-1A cells grown to mid-exponential-phase (OD_600_ of 0.5-0.6) were harvested by centrifugation and suspended in SC galactose (OD_600_ of 0.2) containing 0.1% of DMSO and C2-ceramide (N-acetyl-sphingosine), C6-ceramide (N-hexanoil-sphingosine) or C2-phytoceramide (N-acetyl-D-phytosphyngosine) at the indicated concentrations. Treatments were carried out at 30 °C with agitation (200 r.p.m.). Viability was determined by CFU (colony-forming units) counts after a 2 day incubation on YEPD agar plates at 30 °C. No additional colonies appeared after this period. Results were normalized to O.D. 100% corresponds to the number of CFU at time zero.

### Flow Cytometry

Flow cytometry data acquisition was performed with an Epics XL-MCL (Beckman Coulter) flow cytometer equipped with an argon-ion laser emitting a 488 nm beam at 15 mW. At least twenty thousand cells were analyzed per sample at low flow rate.

### Fluorescence Microscopy

Cells were observed using a Leica Microsystems DM-5000B epifluorescence microscope with appropriate filter settings using a 100x oil-immersion objective. Images were acquired with a Leica DCF350FX digital camera and processed with LAS AF Leica Microsystems software.

### Cell cycle analysis

Cell cycle analysis was performed as described [16] using 1 µM Sytox Green (Molecular Probes). Fluorescence was measured by flow cytometry, and the data was analyzed using FlowJo 7.6 software (Tree Star, Inc).

### Cell synchronization in G0/G1 phase

Yeast cells were grown in SC galactose to mid-exponential phase, and transferred to nitrogen starvation medium (SD–N medium: 0.17% yeast nitrogen base without amino acids and ammonium sulfate, 2% glucose) to an OD_600_ of 0.2. Cells were then incubated at 30 ^°^C for approximately 24 hours (one duplication) and harvested.

### PI staining, ROS accumulation, chromatin condensation assessment and detection of DNA strand breaks

Plasma membrane integrity was assessed by propidium iodide (PI) staining. 10^6^ cells were incubated in culture medium containing 2 µg/ml of PI (Sigma) at room temperature for 10 min, in the dark. Fluorescence was measured by flow cytometry. Cells with red fluorescence [FL-3 channel (488/620 nm)] were considered to have lost plasma membrane integrity. Reactive oxygen species (ROS) accumulation was monitored by flow cytometry using Dihydroethidium (DHE) and other probes (supplemental material). Intracellular generation of superoxide anion was monitored using DHE (Molecular Probes, Eugene, U.S.A.). 1×10^6^ cells were harvested by centrifugation, resuspended in PBS, and stained with 5 µg/ml of dihydroethidium at 30 °C for 30 minutes, in the dark. Fluorescence was measured by flow cytometry. Cells with red fluorescence [FL-3 channel (488/620 nm)] were considered to accumulate superoxide anion. ROS production assessment using dihydrorhodamine 123 (DHR 123), 2′,7′-Dichlorofluorescein diacetate (H_2_DCFDA) and Mitotracker Red CM-H2XRos was performed as previously described [17].

For chromatin condensation assessment, cells were fixed with ethanol, stained with DAPI (4,6-diamidino-2-phenylindole dihydrochloride) and observed by fluorescence microscopy. Viable cells were considered to have very round and clear nuclei whereas apoptotic cells were identified by having smaller, condensed (chromatin gathering at the periphery of the nuclear membrane), fragmented and kidney shaped nuclei. At least 300 cells per sample were counted in three independent experiments and the percentage of apoptotic nuclei determined.

The occurrence of DNA strand breaks was determined by TUNEL assay using the In Situ Cell Death Detection Kit, Fluorescein (Roche Applied Science, Indianapolis, IN) as previously described [18]. Yeast cells treated with 30 µM of C2-phytoceramide and 0.1% DMSO for 120 min were fixed with 3.7% (v/v) formaldehyde. The cell wall was digested with Lyticase, and cells were applied to poly-lysine coated slides. The slides were rinsed with PBS, incubated in permeabilization solution (0.1% v/v, Triton X-100 and 0.1% w/v, sodium citrate) for 2 min on ice, rinsed twice with PBS and incubated with 10 µl TUNEL reaction solution for 60 minutes at 37 ^°^C. Finally, the slides were rinsed three times with PBS and a coverslip was mounted with a drop of anti-fading agent Vectashield (Vector Laboratories, Inc). Slides were analyzed by fluorescence microscopy. Non-treated cells were used as a negative control and DNase I treated cells were used as a positive control for DNA breaks. Green fluorescence indicates TUNEL positive cells. Samples were observed by fluorescence microscopy.

### Sensitivity to Zymolyase

A zymolyase sensitivity assay was performed as described in [19] with modifications. Wild-type yeast cells were cultivated in SC 2% galactose medium with 30 µM of C2-phytoceramide or 0.1% DMSO for 2 h. Cells were then harvested, washed with sterile distilled water and resuspended in 0.1 mM sodium phosphate buffer (pH 7.5). After adding 20 µg/ml of zymolyase (Medac; Medacshop), cell lysis was followed by measuring the decrease in the OD_600_ of each cell suspension.

### Filipin staining

Sterol-lipid distribution was assessed *in vivo* by filipin staining as previously described [20].

### Sensitivity to ergosterol biosynthesis inhibitors, amphotericin B and methy- β-cyclodextrin

W303-1A cells were grown to mid-exponential-phase (OD_600_ of 0.5-0.6), harvested by centrifugation and suspended in SC galactose (OD_600_ of 0.2). Cells were exposed for 30 min to: 300 µM clotrimazole, 300 µM ketoconazole, 5 mg/ml methyl-β-cyclodextrin or 1µg/ml amphotericin B. Afterwards, 0.1% (v/v) DMSO and 30 µM of C2-phytoceramide were added, and cells were again incubated for 120 min at 30 °C with agitation (200 r.p.m.). Viability was determined by CFU counts as described above. All chemicals were obtained at the highest available grade (Sigma-Aldrich).

### Statistical analysis

Two-way ANOVA or One-way with Bonferroni posttest was performed using GraphPad Prism version 5.00 for Windows, GraphPad Software, San Diego California USA (www.graphpad.com). Two-way ANOVA analysis was performed when evaluating two conditions, such as the impact of stress caused by ceramides or DMSO (1^°^ condition) along time (2^°^ condition). One-way ANOVA analysis was performed in experiments evaluating just one condition, usually the stress impact at one time point. The Bonferroni posttest was used because it assumes that the tests are independent of each other.

## Results

### C2-phytoceramide leads to loss of clonogenic survival in *Saccharomyces cerevisiae*

**Figure 1:**
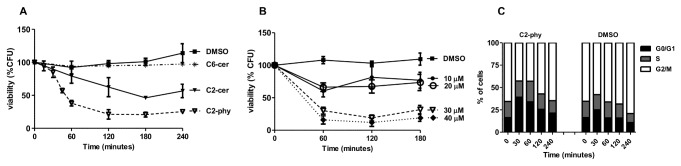
*S. cerevisiae* cells are sensitive to ceramides. (**A**) Survival of W303-1A cells exposed to 30 µM C2-phytoceramide (∇), 30 µM C6-ceramide (*), 30 µM C2-ceramide (▼), or equivalent volume of solvent (■). CFU values of C2-treated cells significantly different from DMSO-treated cells, P<0.0001, Two-Way ANOVA. (**B**) Survival of W303-1A cells exposed to 10 µM (●), 20 µM (○), 30 µM (∇-dashed lines) and 40 µM (♦-dashed lines) C2-phytoceramide, or equivalent volume of solvent (■). CFU values of C2-phytoceramide-treated cells (30 µM or 40 µM) are significantly different from DMSO-treated cells for all time points, P<0.001, Two-Way ANOVA. All CFU values (A and B) represent mean ± SE of at least 3 independent experiments, with 5 replicas in each experiment. (**C**) Cell cycle progression of cells exposed to 30 µM C2-phytoceramide or equivalent volume of solvent. Data from a representative experiment (of 3 independent experiments) is shown.

Phytoceramides, the yeast counterparts of mammalian ceramides, mediate regulation of cell growth and stress responses in yeast. Exposure of mammalian cell lines to C2-ceramide mimics the effect of ceramide generation in response to chemotherapeutic drugs or other stress conditions [3]. In order to explore yeast as a model system to further understand the molecular basis of ceramide-induced effects, we tested whether exogenously added phytoceramides, like ceramides in mammalian cells, could induce cytotoxicity in yeast. Clonogenic survival was assessed in *Saccharomyces cerevisiae* W303-1A cells exposed to the soluble and cell-permeable phytoceramide N-acetyl-phytosphingosine (C2-phytoceramide), N-acetyl-sphingosine (C2-ceramide) or N-hexanoil-sphingosine (C6-ceramide) for up to 240 min. C2-ceramide or C2-phytoceramide decreased cell clonogenic survival, but CFU counts of cells exposed to C6-ceramide were indistinguishable from those of DMSO-treated control cells (Figure 1A). C2-phytoceramide led to the highest decrease in CFU, which was dose-dependent in the range of 10 to 40 µM and started to be rapidly observed (Figure 1A, 1B). A similar sensitivity to C2-phytoceramide was also observed with another *S. cerevisiae* strain background, showing that the effect was not specific for W303-1A (Figure S1).

**Figure 2:**
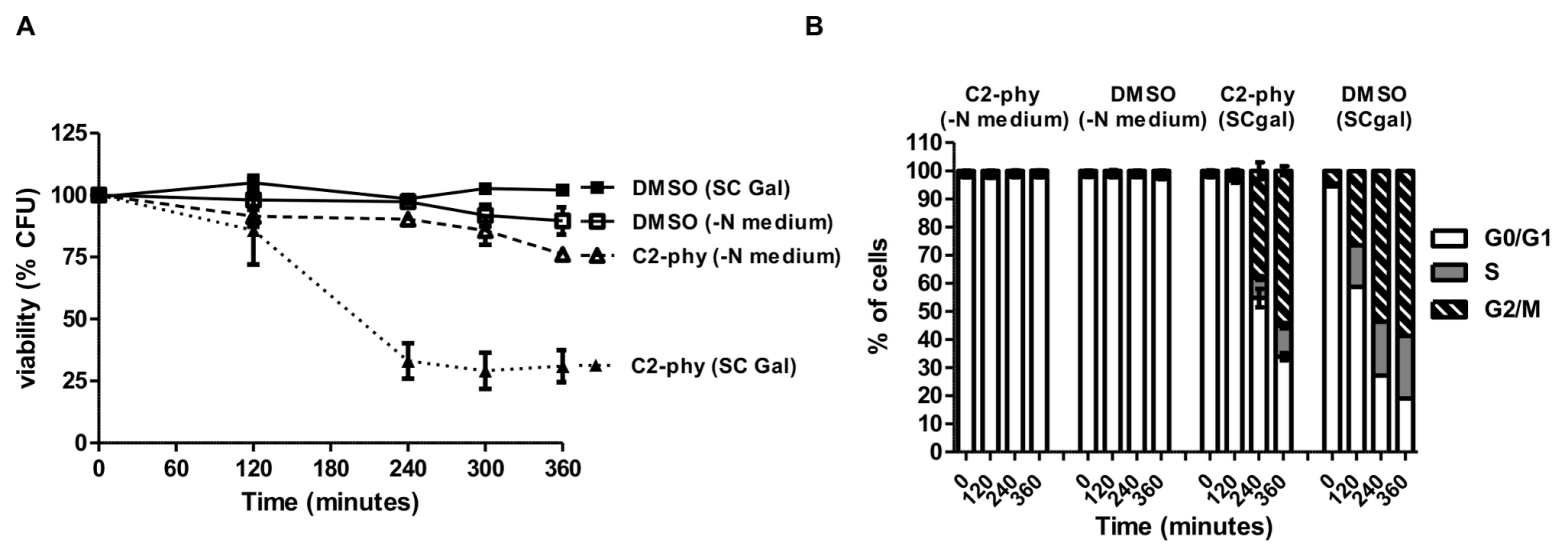
*S. cerevisiae* cells in G2/M are more sensitive to C2-phytoceramide. (**A**) Survival of W303-1A cells synchronized in nitrogen starvation medium. Cells were transferred to SC gal medium and then exposed to 30 µM C2-phytoceramide (▼-dashed lines) or equivalent volume of solvent (■), or maintained in nitrogen starvation medium and exposed to 30 µM C2-phytoceramide (∇-dashed lines) or equivalent volume of solvent (□). CFU values of cells transferred to SC gal and treated with 30 µM C2-phytoceramide are significantly different from C2-phytoceramide treated cells kept in nitrogen starvation medium and from DMSO-treated cells transferred or not to SC Gal, for all time points, P<0.001, Two-Way ANOVA. All CFU values represent mean ± SE of at least 3 independent experiments, with 5 replicas in each experiment. (**B**) Cell cycle progression of the cells described in (A). Data represent mean ± SE of at least 3 independent experiments.

Next, we questioned whether C2-phytoceramide, as described for C2-ceramide [7], could alter cell cycle progression. C2-phytoceramide led to an accumulation of cells in the G0/G1 phase (Figure 1C), similarly to that described for C2-ceramide. To address whether C2-phytoceramide-induced loss of CFU was cell-phase specific, cells were synchronized in G0/G1 by incubation under nitrogen starvation for 24 hours prior to treatment. G0/G1-synchronised cells were then harvested, centrifuged and resuspended in the same starvation medium or in liquid synthetic complete medium (SC), with C2-phytoceramide (dissolved in DMSO) or with the same amount of DMSO. Cells that were kept in the starvation medium, and so not release from G0/G1 arrest, were not sensitive to C2-phytoceramide (Figure 2A). In cells transferred to SC medium with C2-phytoceramide, CFU started to decrease only when a significant percentage of the population proceeded to the G2/M phase, and was thus delayed relatively to unsynchronized cells (Figure 2A and 2B). These results indicate that C2-phytoceramide-induced decrease in CFU occurs preferentially in dividing cells.

### C2-phytoceramide does not elicit an apoptotic phenotype or trigger autophagy

**Figure 3:**
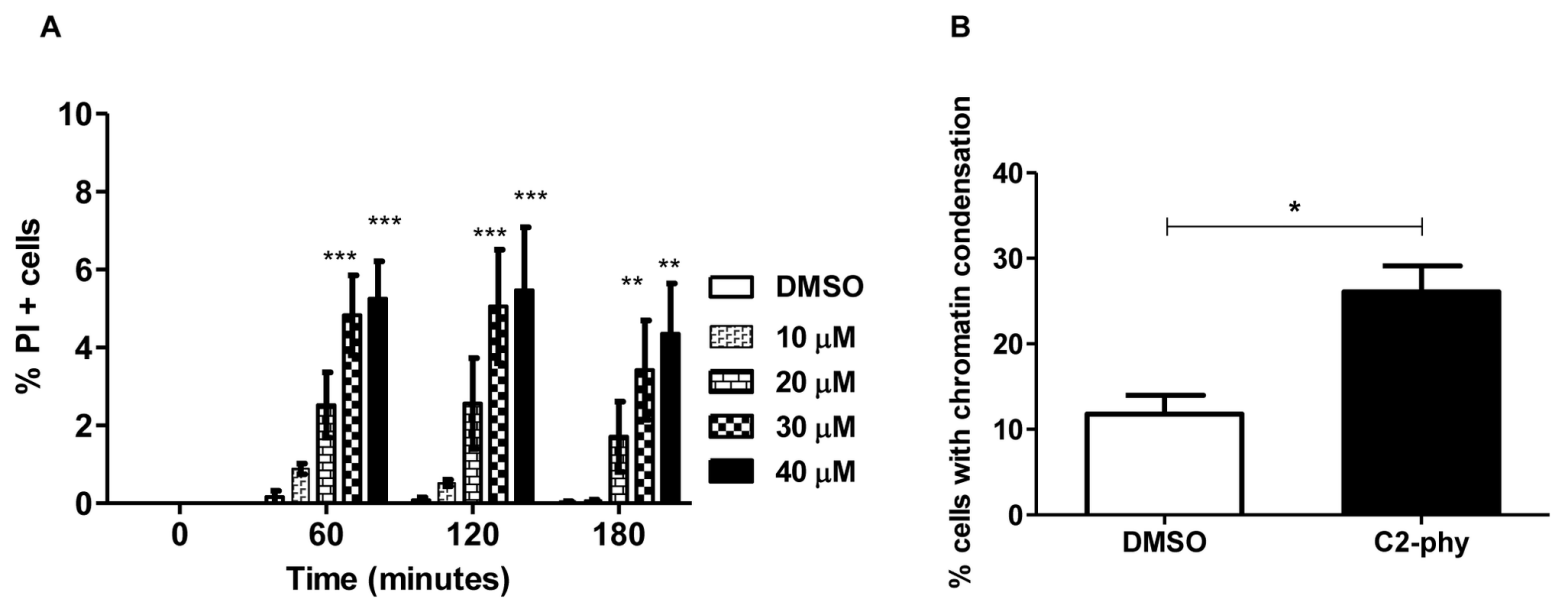
Cell death markers observed after exposure of *S. cerevisiae* to C2-phytoceramide. (**A**) Percentage of PI positive cells in W303-1A cultures exposed to C2-phytoceramide or to equivalent volume of solvent. Values represent mean ± SE of at least 3 independent experiments. Percentages of PI-stained cells of C2-phytoceramide-treated cells (30 µM and 40 µM) significantly different from DMSO-treated cells, P<0.001 for time 60 and 120 min and P<0.01 for time 180 min, Two-Way ANOVA. (**B**) Percentage of yeast cells with chromatin condensation, determined by counting at least 300 cells per sample after a 120 min treatment with 30 µM C2-phytoceramide or equivalent volume of solvent, in three independent experiments. *P*<0.05 One-Way ANOVA.

It had been shown that C2-ceramide induces mitochondrial-dependent apoptotic and necrotic phenotypes in *S. cerevisiae* BY4741 [13]. We therefore monitored cell death markers after exposure to C2-phytoceramide. Exposure to C2-phytoceramide did not lead to DNA fragmentation assessed by TUNEL staining (Figure S2) and only slightly increased the percentages of cells with positive propidium iodide (PI) staining or with nuclear chromatin condensation (15%) visualized after DAPI staining (Figure 3). Moreover, C2-phytoceramide did not elicit mitochondrial alterations, including relevant ROS accumulation assessed by staining with DHE, H_2_DCFDA/IP, DHR 123 or Mitotracker Red (Figure S3), nor mitochondrial fragmentation or degradation assessed using cells expressing fluorescent mtGFP (Figure S4). Accordingly, C2-phytoceramide-induced loss of CFU could not be rescued by simultaneously treating cells with ROS scavengers (Figure S5A), or by overexpressing the anti-apoptotic protein Bcl-X_L_ (Figure S5B), suggesting that mitochondrial function was not involved in C2-phytoceramide-induced loss of CFU. In agreement with this hypothesis, the sensitivity of a mitochondrial-deficient mutant strain (*rho* ^*o*^) to C2-phytoceramide was indistinguishable from that of the wild type strain (Figure S5C). Deletion of the yeast metacaspase *YCA1* also had no effect on the response to C2-phytoceramide, and consistently caspase activation assessed by Z-VAD-FITC/IP double staining was not detected (Figure S6). Taken together, these results show that C2-phytoceramide does not induce an apoptotic pathway in *S. cerevisiae* W303-1A cells.

Autophagy is a physiological process by which cells sequester and degrade bulk cytosol and organelles for recycling. It is important for the survival of cells under nitrogen starvation conditions, but also has a housekeeping function in removing damaged, redundant, or unwanted cellular components [21]. In mammalian cells, autophagy can be triggered by C2-ceramide, involving AKT/PKB, mTOR-signaling and dissociation of the Beclin 1: Bcl-2 complex after c-Jun N-terminal kinase 1 (JNK1)-dependent Bcl-2 phosphorylation [22]. Under specific conditions and depending on cell type and context, autophagy has also been proposed to mediate cell death [22]. We therefore evaluated whether C2-phytoceramide induced autophagy in wild-type cells, possibly contributing to C2-phytoceramide-induced loss of CFU in *S. cerevisiae*. We measured cleavage of Atg8-GFP, a commonly used marker of autophagy, and also assessed the kinetics of C2-phytoceramide-induced loss of CFU in the autophagy-defective mutant *atg5*Δ. Exposure of wild-type cells to C2-phytoceramide did not lead to Atg8-GFP cleavage (Figure S7A) and, consistently, absence of Atg5p did not rescue cells from C2-phytoceramide induced loss of CFU (Figure S7B), thus excluding a role for autophagy in this process.

### Cell integrity defects underlie C2-phytoceramide-induced loss of CFU

**Figure 4:**
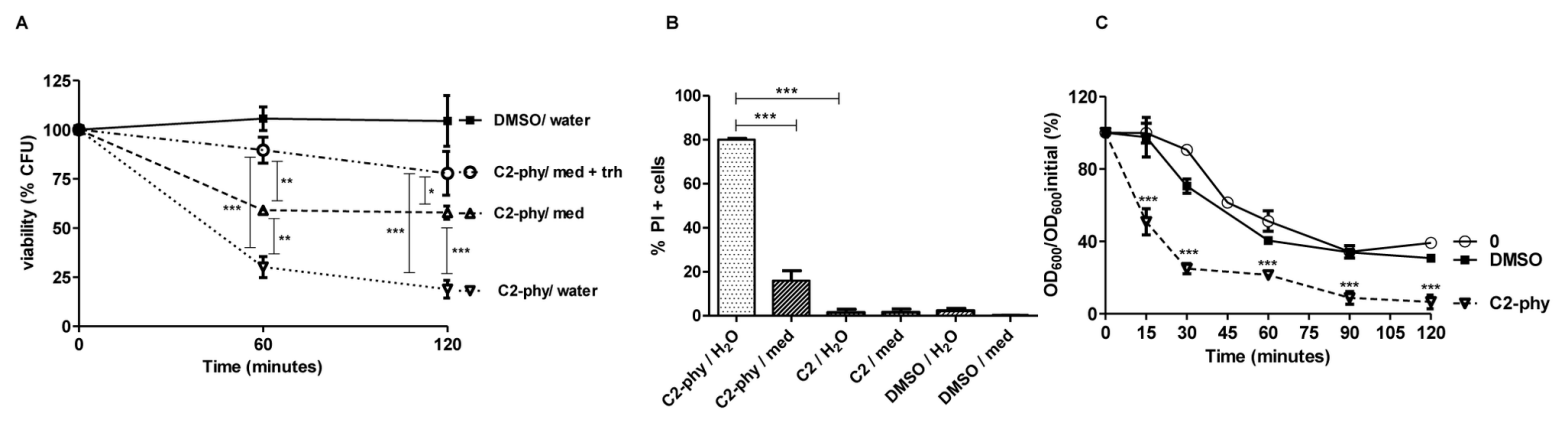
C2-phytoceramide sensitizes *S. cerevisiae* to hyposmotic stress and leads to defects in cell wall integrity. (**A**) Survival of W303-1A cells exposed to 30 µM C2-phytoceramide or equivalent volume of solvent (■). Before plating on solid media, cells were diluted in water (∇), SC medium (Δ), or SC medium with 2% trehalose (○). Data are given as mean ± SE of 3 independent experiments, with 5 replicas in each experiment. CFU values of C2-phy treated cells diluted in water were significantly different from C2-phy treated cells diluted in medium + trehalose, P<0.001, Two-Way ANOVA. Viability of C2-phy treated cells diluted in water were significantly different from C2-phy treated cells diluted in medium, P<0.01 for time 60 min and P<0.001 for time 120 min, Two-Way ANOVA. CFU values of C2-phy treated cells diluted in medium were significantly different from C2-phy treated cells diluted in medium + trehalose, P<0.01 for time 60 min and P<0.05 for time 120 min, Two-Way ANOVA (**B**) Percentage of P.I. positive cells, after treatment with 0.1% DMSO, 30 µM of C2-phytoceramide, or 40 µM of C2-ceramide for 120 min. Cells were diluted 100x in water and in SC medium and then stained with 2 µg/ml PI for 10 min in the dark. *P*<0.001 One-Way ANOVA. (**C**) Sensitivity of W303-1A cells exposed to 30 µM C2-phytoceramide (∇), 40 µM C2-ceramide (▲) or equivalent volume of solvent (☐) to digestion with zymolyase. Non-treated cells were used as a control (○). Values represent mean ± SE of 3 independent experiments. C2-phytoceramide treated cells were significantly different from non-treated and DMSO treated cells, P<0.001, Two-Way ANOVA.

Since there was no significant increase in apoptotic or autophagic markers in cultures treated with C2-phytoceramide, we questioned if hypo-osmotic stress associated with dilution of the samples in sterile water before plating, a manipulation performed only for clonogenic assays, was responsible for the decrease in CFU. Diluting cells in culture medium instead of sterile water before plating indeed partially prevented the loss of clonogenic survival, and this protection increased if the osmoprotector trehalose was added to the dilution medium (Figure 4A). In addition, in contrast with cells diluted in medium, diluting the cells in water led to a massive permeabilization to PI, indicative of necrosis (Figure 4B). This effect was specific to C2-phytoceramide, as there was no difference in membrane permeability to PI of cells exposed to C2-ceramide either when diluted in water or in medium (Figure 4B). Exposure of cells to C2-phytoceramide also led to increased susceptibility to digestion with the cell wall-degrading enzyme zymolyase, which again was not observed for C2-ceramide, suggesting that defects in cell wall integrity underlie C2-phytoceramide-induced loss of CFU (Figure 4C). Since C2-phytoceramide effects were particularly evident in cells in the G2/M phase (Figure 2), these results support the idea that C2-phytoceramide perturbs cell wall assembly. We therefore next studied how C2-phytoceramide affects lipid rafts, as defects in these plasma membrane structures can perturb cell wall assembly [23].

### C2-phytoceramide perturbs ergosterol distribution in lipid rafts

**Figure 5:**
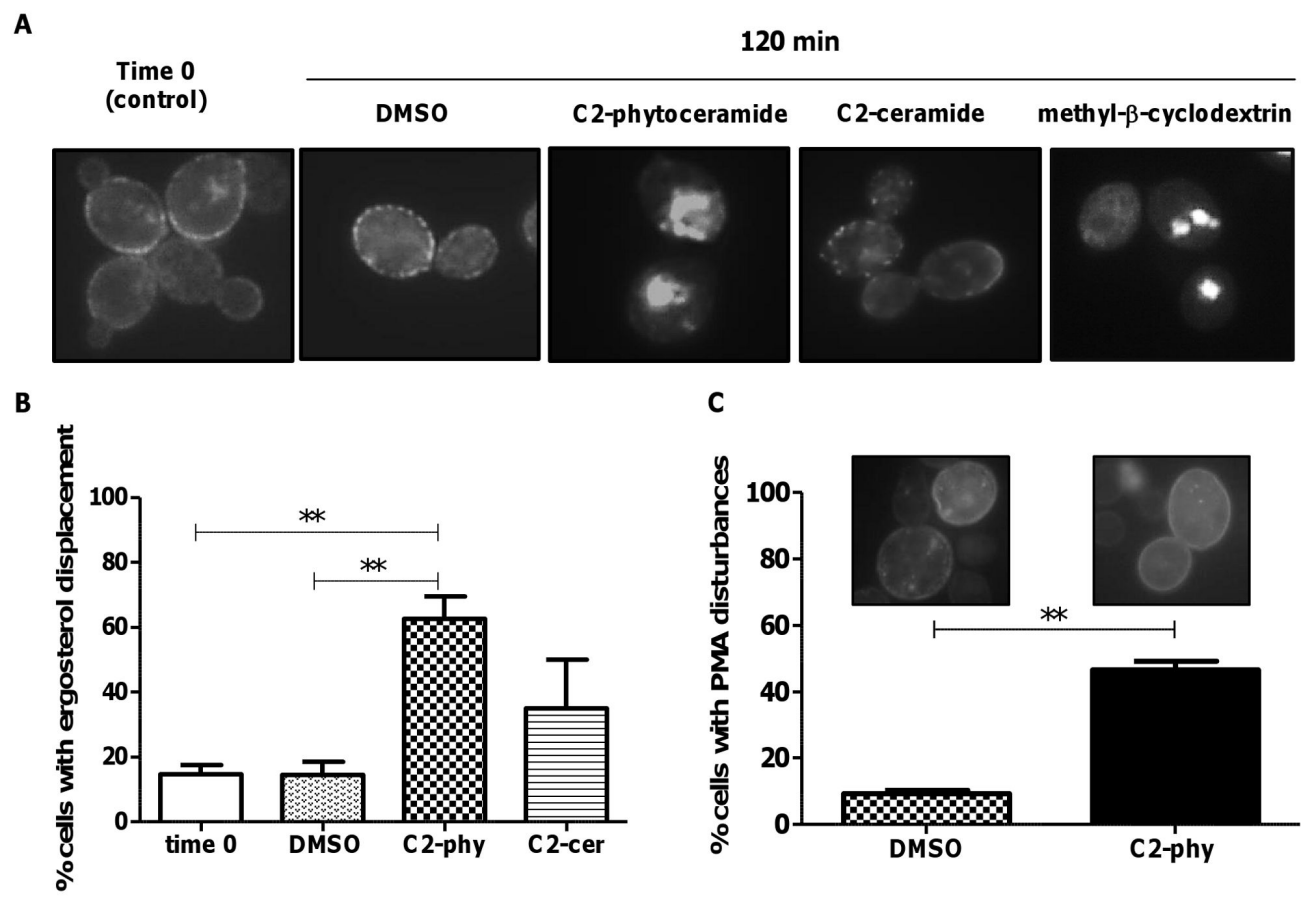
Distribution of sterol-rich domains in *S. cerevisiae* cells exposed to C2-phytoceramide. (**A**) Fluorescence microscopy pictures of W303-1A cells exposed to 30 µM C2-phytoceramide, 40 µM C2-ceramide, 5 mg/ml methyl-®-cyclodextrin or 0.1% DMSO for 120 min and stained with filipin (5 mg/ml). (**B**) Percentage of yeast cells with ergosterol displacement. Cells were treated as described in (**A**) and the number of cells with ergosterol displacement was determined by counting at least 120 cells per sample, in three independent experiments. P<0.01 respectively, One-Way ANOVA. (**C**) Percentage of yeast cells with perturbed Pma1p-GFP distribution. W303-1A cells were transformed with a single copy vector derived from pRS316 expressing Pma1p-GFP (3). At least 300 cells per sample were counted. P<0.01.

**Figure 6:**
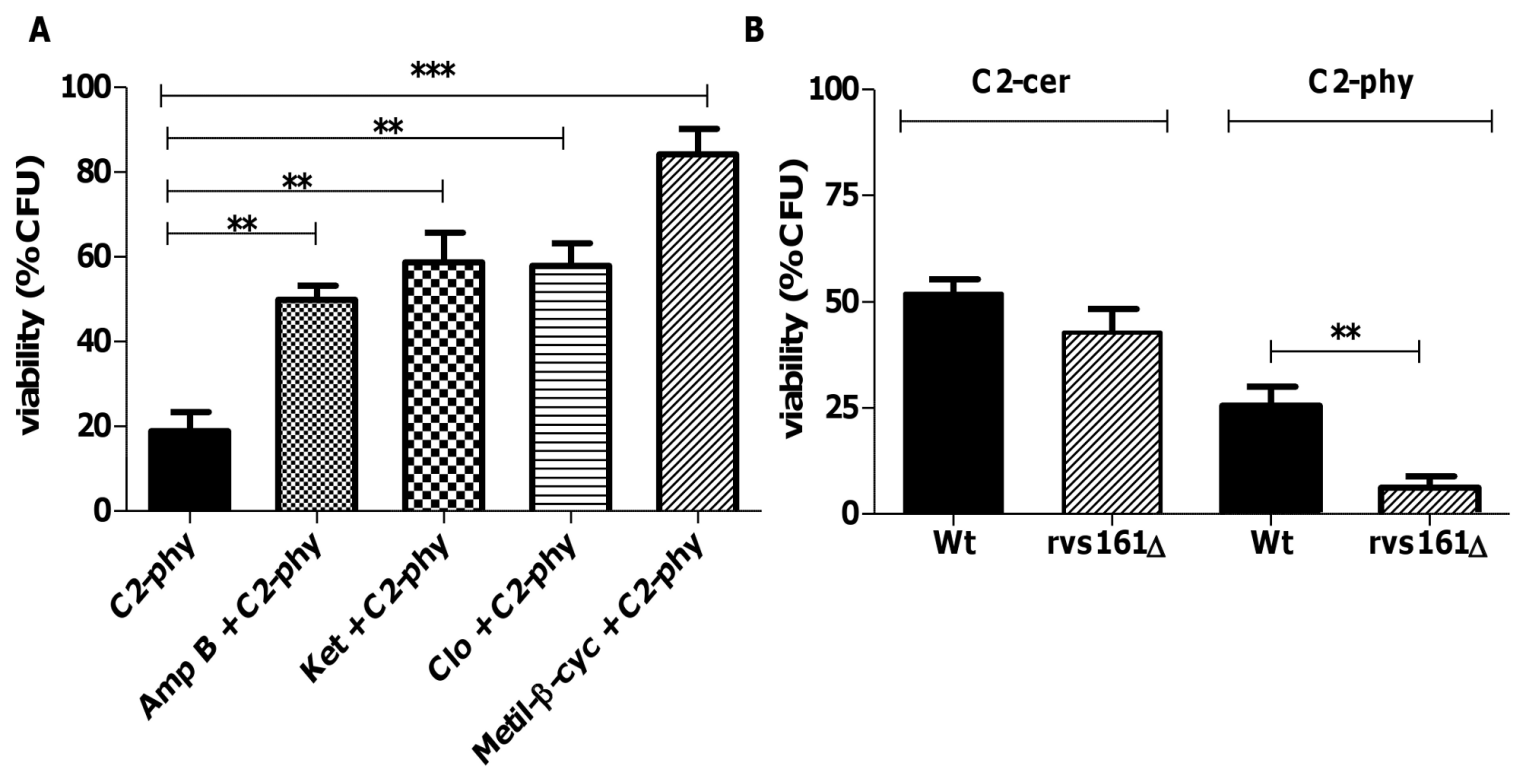
Loss of cell viability is rescued by ergosterol biosynthesis inhibitors, amphotericin B and methyl-β-cyclodextrin. (**A**) Survival of W303-1A cells exposed to amphotericin B 1 µg/ml, ketoconazole 300 µM, clotrimazole 300 µM or methyl-β-cyclodextrin 5 mg/ml for 30 min followed by an incubation with C2-phytoceramide for 120 min. Values represent mean ± SE of at least 3 independent experiments, with 5 replicas in each experiment. (**B**) Survival of wild type W303-1A and *rvs161*Δ cells exposed to 30 µM C2-phytoceramide or 30 µM C2-ceramide. CFU values of the C2-phytoceramide-treated mutant cells are significantly different from wild type-treated cells P<0.01, One-Way ANOVA.

Rafts are membrane lipid microdomains formed by lateral association of sphingolipids and ergosterol in yeasts, indispensable for the anchoring of proteins responsible for cell wall biogenesis and assembly [24]. They play fundamental roles in connecting the plasma membrane to the cytoskeleton and endoplasmic reticulum and Golgi apparatus, i.e., for correct protein sorting and trafficking through exocytosis/endocytosis [24]. To test whether defects in cell integrity were a consequence of an alteration in lipid rafts mediated by C2-phytoceramide, we monitored cellular ergosterol distribution using filipin, a polyene antibiotic with fluorescent properties that binds sterols. The characteristic dot staining of rafts at the plasma membrane were decreased in C2-phytoceramide-treated cells, while staining of intracellular structures was increased; C2-ceramide had no effect on filipin distribution (Figure 5A,B). To confirm that the decreased plasma membrane staining corresponded to a decrease in ergosterol content, we treated cells with methyl-β-cyclodextrin, which extracts ergosterol from membranes [25]. Treatment with this compound yielded a similar filipin-staining pattern as C2-phytoceramide (Figure 5A). Furthermore, pre-treatment of cells with inhibitors of ergosterol biosynthesis, with methyl-β-cyclodextrin, or with the ergosterol-binding antibiotic amphothericin B, increased resistance to C2-phytoceramide (Figure 6A). Taken together, these results indicate that exposure of *S. cerevisiae* cells to C2-phytoceramide leads to a perturbation in the sterol-rich membrane micro-domains known as lipid rafts and suggest ergosterol is the target of C2-phytoceramide. The perturbation of lipid rafts was also evaluated by observing the distribution of Pma1p, the plasma membrane ATPase that localizes in these structures. C2-phytoceramide led to a uniform rather than punctuated pattern of a GFP-tagged version of Pma1p at the plasma membrane in a high percentage of cells (Figure 5C).

We next assessed the impact of perturbing lipid rafts on the response to C2-phytoceramide using the *rvs161*Δ mutant. The Rvs161 protein is localized in lipid rafts and is involved in cytoskeleton organization, cell polarity and cell wall synthesis, as well as in cell survival following osmotic stress [26]. Absence of Rvs161p resulted in increased sensitivity to C2-phytoceramide, but not to C2-ceramide (Figure 6B). These results confirm the involvement of raft-mediated processes in the loss of CFUs induced by C2-phytoceramyde during cell growth. Since Rvs161p is involved in cytoskeleton organization, we questioned if C2-phytoceramide induces alterations in actin organization. However, treatment with this compound did not result in an altered pattern of rhodamine phalloidin staining (Figure S8).

### The HOG pathway increases resistance to C2-phytoceramide

**Figure 7:**
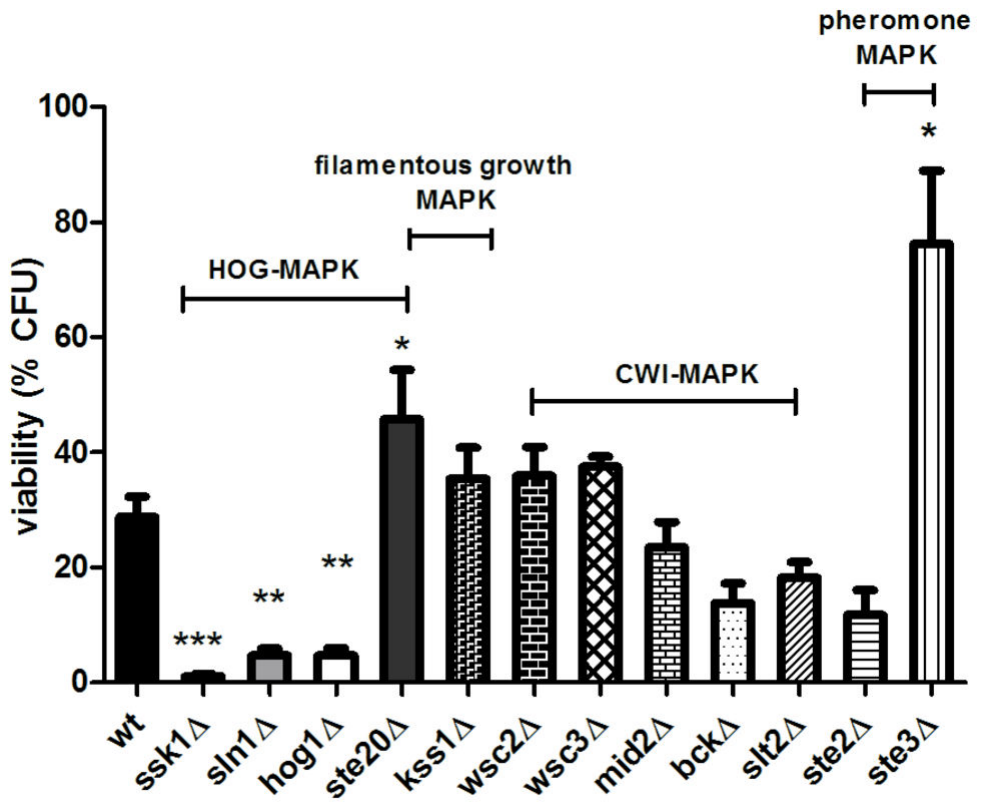
The MAP kinase pathways are involved in the sensitization of *S. cerevisiae* to C2-phytoceramide. Survival of wild type W303-1A cells and of mutant strains after exposure to 30 µM C2-phytoceramide for 120 min. (*ssk1*Δ, P<0.0001, *sln1*Δ, P<0.0001 *hog1*, P<0.01 and *ste20*Δ, P<0.05), (*ste3*Δ, P<0.05), One-Way ANOVA. Data are given as mean ± SE of at least 3 independent experiments, with 5 replicas in each experiment.

Two Mitogen Activated Protein Kinase (MAPK) pathways of the yeast *Saccharomyces cerevisiae* are important in the response to osmotic stress, the cell wall integrity (CWI) pathway, essential for the response to hypo-osmotic stress, and the High Osmolarity Glycerol (HOG) response pathway. We therefore tested the response of strains deficient in different components of these two pathways to C2-phytoceramyde. *slt2*Δ, *wsc2*Δ, *wsc3*Δ, *mid2*Δ and *bck1*Δ cells, deficient in components of the CWI pathway, displayed no differences in response to this compound (Figure 7), suggesting that the CWI pathway does not signal sensitization to osmotic stress in response to C2-phytoceramide.

The HOG pathway has a central role in resistance and adaptation to osmotic stress, through both transcriptional and non-transcriptional responses [27]. We tested mutant strains lacking the Hog1p MAPK, ortholog of the human JNK and p38, and components of the two upstream signaling branches, namely, Sln1p, Ssk1p and Ste20p. *hog1*Δ, *sln1*Δ and *ssk1*Δ mutant strains were more sensitive to C2-phytoceramide than wild-type cells, whereas the *ste20*Δ strain was more resistant (Figure 7), indicating that the Sln1p branch of the HOG pathway, but not the Sho1p branch, is important for resistance to C2-phytoceramide-induced loss of CFU. It has been reported that perturbation of raft structure by filipin impedes activation of the Sho1p branch [28]. This may explain the involvement of Sln1p branch, rather than Sho1p, in the response to C2-phytoceramide, which we also found disturbs lipid rafts.

The Ste20p kinase is a component of three signaling cascades in yeast that control the osmoregulatory response to hypertonic stress (HOG pathway), the pheromone response, and filamentous growth pathways. The involvement of Ste20p in these last two pathways could explain the resistance phenotype of the deletion mutant. Absence of the G-protein–coupled yeast a-pheromone receptor Ste3p increased resistance to C2-phytoceramide, while deficiency in the -pheromone receptor Ste2p and in Kss1p, a downstream component of the filamentous growth pathway, did not affect this response (Figure 7). Taken together, these results suggest that the *ste20*Δ resistance phenotype may stem from its involvement in the pheromone response pathway.

Since our results show that Hog1p is important for the resistance to C2-phytoceramide, we tested if this compound could induce Hog1p activation. There was a moderate Hog1p phosphorylation, at time zero, indicating that the Hog pathway was already activated under our experimental conditions. After 5 min of incubation with C2-phytoceramide there was a slight increase in Hog1p phosphorylation, though not statistically different when compared with vehicle DMSO-treated control cells (Figures S9A and S9B). Consistently with Hog1 basal activation, pre-incubation with sorbitol before treatment with C2-phytoceramide did not affect cell survival (Figure S9C). The results indicate that the presence of active Hog1p is responsible for promoting resistance to osmotic stress and are consistent with the sensitive phenotype of the *hog1*Δ mutant.

## Discussion

In the last decades, ceramides have been implicated in the regulation of cell proliferation and cell death in both mammalian and yeast cells [29]. Therefore, interfering with ceramide metabolism or direct exposure to ceramides have been explored as potential therapeutic strategies for diseases associated with dysfunctional cell fate regulation [30]. In this study, we aimed to explore yeast as a model system to unravel the still poorly understood mechanisms and signaling pathways underlying ceramide cytotoxicity. We characterized the effect of C2-phytoceramide, a yeast counterpart of mammalian ceramides, on the viability of *Saccharomyces cerevisiae* W303-1A cells. In accordance with a previous study with SK–N-BE(2) C and N1E-115 mammalian cell lines [31], C2-phytoceramide led to a decrease in CFU, but not to typical apoptotic markers. Accordingly, C2-phytoceramide cytotoxicity could not be inhibited by ROS scavengers or by overexpression of the anti-apoptotic protein Bcl-xL. In agreement with the lack of caspase activation, absence of the yeast metacaspase Yca1p did not rescue cells from C2-phytoceramide cytotoxicity. This is in accordance with the previously described caspase-independent cell death induced by C2-ceramide in *S. cerevisiae* BY4741 cells [13], although our results show that cytotoxicity induced by C2-phytoceramide differs significantly from that of C2-ceramide, namely because it does not depend on mitochondrial function.

It was reported that ceramide induces autophagy in mammalian cells by interfering with different signaling pathways [22]. Therefore, we hypothesized that excessive autophagy could underlie the observed sensitive phenotype. However, C2-phytoceramide did not trigger autophagy and sensitivity was not affected in the absence of Atg5p. A previous report indicating that the yeast proteins Ipt1p and Skn1p, involved in the biosynthesis of mannosyldiinositolphosphoryl ceramides, negatively regulate autophagy induced by nitrogen (N) starvation [32] may explain our results.

C2-phytoceramide, as previously described for C2-ceramide [7], affects yeast cell cycle progression, inducing a delay in G0/G1. We found that sensitivity to C2-phytoceramide was also cell cycle-specific, as it was observed preferentially in G2/M phase cells. This observation is consistent with a report showing that pro-B-cell lymphoid cultures (FL5.12) growing in the presence of high levels of IL-3 growth factor are more sensitive to ceramide than cells maintained in low levels of this compound [33]. It would also explain why cancer cells, usually dividing more actively, are more sensitive to ceramide than non-dividing cells.

Our results indicate that exposure of cells to C2-phytoceramide causes defects in the cell wall, rendering them more sensitive to digestion with zymolyase. This sensitization was not due to nonspecific detergent-like effects on the plasma membrane, as it was not observed for the structurally close C2-ceramide. Rather, it appears to be associated with defects in ergosterol distribution and lipid rafts. This is in line with previous reports using lipid vesicles containing co-existing raft domains and disordered fluid domains, which showed that ceramides displaced cholesterol from rafts while other raft lipids remained raft-associated in the presence of ceramides [34]. Filipin staining of C2-phytoceramide-treated cells and the resistance of ergosterol-depleted cells to C2-phytoceramide support that C2-phytoceramide may have a similar effect on yeast lipid rafts, displacing ergosterol, the yeast equivalent of mammalian cholesterol. Two other observations support this hypothesis: the sensitive phenotype of the *rvs161*Δ strain and the C2-phytoceramide-induced change in the distribution of the plasma membrane ATPase Pma1p from its usual punctuated pattern to a uniform distribution. Disruption of these structured membrane microdomains may compromise different essential cell functions, including cell wall and plasma membrane synthesis in dividing yeast cells. Moreover, it was recently shown that components of lipid rafts, namely ergosterol, regulate the HOG pathway [35]. Accordingly, absence of Hog1p drastically increased cell susceptibility, reinforcing that C2-phytoceramide cytotoxicity can be counterbalanced by the protection against osmotic stress afforded by Hog1p. Hog1p is also involved in the inhibition of ergosterol biosynthesis in response to hypoxia stress [36]. The higher sensitivity of the *hog1*Δ mutant might therefore be a result of an increase in ergosterol content in this strain, as we also observed that inhibiting ergosterol biosynthesis increased resistance to C2-phytoceramide.

Absence of the pheromone response pathway components Ste3p and Ste20p resulted in higher resistance to C2-phytoceramide-induced loss of CFU. These results are consistent with the observed G1 cell cycle arrest and weakening of the cell wall induced by C2-phytoceramide, since these are two well-characterized effects associated with the activation of the pheromone response pathway [37]. This pathway has been implicated in necrotic cell death in response to pheromone [37], and our results suggest that it can also be involved in the mediation of necrotic cell death in response to C2-phytoceramide. However, the involvement of this pathway needs further clarification as *STE3* deletion is not expected to have a phenotype in a mating type **a** (Mata) strain, where Ste3p expression is repressed [38].

Since lipid rafts are also important in signaling and the organization of the cell wall-like extracellular matrix in mammalian cells, the results presented here suggest that different effects of ceramide previously described, such as anoikis induction in HeLa cells [39] or altered signaling described for many cell systems, may be associated with a similar disorganization of lipid rafts. This study identified a new function of C2-phytoceramide through interference with cell wall stability, likely mediated by the disorganization of lipid rafts. Our results also substantiate that manipulation of MAPK pathways or changes in sterol content or availability at the plasma membrane can modulate sensitivity to C2-phytoceramide. Since breast and prostate cancer cell lines contain more cholesterol-rich lipid rafts than their normal counterparts [40], our results showing that high levels of sterols render cells more sensitive to C2-phytoceramide suggest that cancer cells can be potential preferential targets for this compound. Furthermore, pharmacological modulation of the conserved MAPK pathways involved in ceramide signaling can also result in increased sensitivity to C2-phytoceramide. As a whole, our results further support pursuing the use of yeast cells as a model system to elucidate the effects of synthetic ceramides and provide improved understanding of the molecular basis of their action with potential implications in the discovery of novel strategies for therapeutic intervention in different diseases.

## Supporting Information

Figure S1 ***S. cerevisiae* W303-1A and BY4741 cells are sensitive to C2-phytoceramide.** Survival of W303-1A (full lines) and BY4741 (dashed lines) cells exposed to 30 µM C2-phytoceramide (●,♦) or equivalent volume of solvent (■, ▲). CFU values of C2-treated cells significantly different from DMSO-treated cells, P<0.001, Two-Way ANOVA. All CFU values represent mean ± SE of at least 3 independent experiments, with 5 replicas in each experiment.
(PDF)

Figure S2 **C2-phytoceramide does not induce DNA fragmentation assessed by TUNEL.** **A**. Fluorescence images of TUNEL staining of *S. cerevisiae* W303-1A treated with 30 µM of C2-phytoceramide or 0.1% (v/v) DMSO for 120 min. Non-treated cells were used as a negative control and DNase I treated cell were used as a positive control for DNA breaks. **B**. Fluorescence images of TUNEL staining of *S. cerevisiae* W303-1A treated with 180 mM of acetic acid for 150 min, used as a positive control for induction of DNA breaks during an apoptotic process [41]. The occurrence of DNA strand breaks was determined using the In Situ Cell Death Detection Kit, Fluorescein (Roche Applied Science, Indianapolis, IN) as previously described [18].
(PDF)

Figure S3 **C2-phytoceramide does not lead to significant ROS accumulation.** ROS production in *S. cerevisiae* W303-1A cells exposed to 30 µM C2-phytoceramide or equivalent volume of solvent (0.1% v/v, DMSO) for up 120 min was measured by flow cytometry using: **A**. dihydroethidium (DHE): Differences between C2-phytoceramide and DMSO treated cells are not significant, P > 0.05 (ns) One-Way ANOVA. Data are given as mean ± SE of at least 3 independent experiments, with 5 replicas in each experiment; **B**. dihydrorhodamine 123 (DHR 123): For T0, P > 0.05 (ns), T60, P<0.001 and for T120, P<0.01. Two-Way ANOVA. Data are given as mean ± SE of 3 independent experiments; **C**. 2′, 7′-Dichlorofluorescein diacetate (H _2_DCFDA): Differences between C2-phytoceramide and DMSO treated cells are not significant, P > 0.05 (ns) Two-Way ANOVA; D. Mitotracker Red CM-H2XRos :. For T0 and T60, P>0.05 (ns), and for T120, P<0.05. Two-Way ANOVA. Data are given as mean ± SE of 3 independent experiments. Intracellular generation of superoxide anion was monitored using DHE, (Molecular Probes, Eugene, U.S.A.). 1×10^6^ cells were harvested by centrifugation, resuspended in PBS, and stained with 5 µg/ml of dihydroethidium at 30 °C for 30 minutes, in the dark. Fluorescence was measured by flow cytometry. For detection of intracellular ROS with dihydrorhodamine 123 (DHR123) (Molecular Probes, Eugene, OR, USA) a 2.5 mg/ml stock solution in DMSO was added to 10^6^ cells/ml suspended in PBS, to a final concentration of 15 µg/ml. Cells were incubated at 30°C for 90 minutes, in the dark. Results are expressed as ratio values estimated by dividing the mean fluorescence intensity of each sample by the mean fluorescence intensity of time zero. For detection of ROS with H _2_DCFDA (Molecular probes), a double staining protocol with PI was used. Conversion of H _2_DCFDA to DCF was analyzed in PI negative cells. 10^6^ cells were incubated in culture medium containing 40 µg/ml H_2_DCFDA at 30 °C for 45 min, in the dark. 2 µg/milliliter of PI was added after 30 min of incubation. For detection of ROS with Mitotracker Red CM-H2XRos (Molecular Probes, Eugene, OR) 0.5x10^7^ cells were resuspended in 500 µL of PBS with 1 mM of the probe, and incubated at 37 °C for 20 min, in the dark.
(PDF)

Figure S4 **C2-phytoceramide does not induce mitochondrial fragmentation and degradation.** **A**. Mitochondrial morphology of *S. cerevisiae* W303-1A cells expressing mitochondrial targeted GFP (W303-1A transformed with pYES2-mtGFP), treated with 30 µM C2-phytoceramide or with 0.1% (v/v) DMSO for 120 min. Non-treated cells (time 0) were used as a control. **B**. Quantification of the percentage of cells displaying GFP fluorescence over the treatment described in (A). Loss of GFP fluorescence was used as a measure of mitochondrial degradation. Values are mean ± SE of 3 independent experiments.
(PDF)

Figure S5 **Loss of cell viability induced by C2-phytoceramide could not be inhibited by 20 µM of N-acetylcysteine (NAC), by overexpression of the anti-apoptotic protein Bcl-xL or in a *rho*0 mutant.** **A**. Survival of *S. cerevisiae* W303-1A cells exposed to 30 µM C2-phytoceramide (○), 30 µM C2-phytoceramide and 20 µM NAC (●), 0.1% (v/v) DMSO (□) and 0.1% (v/v) DMSO with 20 µM NAC (■) for up to 120 min. **B**. Survival of *S. cerevisiae* W303-1A cells overexpressing Bcl-xL, exposed to 30 µM C2-phytoceramide (▼) or 0.1% (v/v) DMSO (●) for up to 180 min. W303-1A cells harboring the empty plasmid (pYES2) treated with 30 µM C2-phytoceramide (○) or 0.1% (v/v) DMSO (□). **C**. Survival of *S. cerevisiae* W303-1A cells (∇) and *rho*0 mutant (*) exposed to 30 µM C2-phytoceramide for up to 120 min. In all experiments 100% corresponds to the number of CFU at time zero. Values are means ± SE of 3 independent experiments.
(PDF)

Figure S6 **Caspases activation and Yca1p are not involved in C2-phytoceramide-induced loss of CFU.** **A**. Caspase activation assessed by flow cytometry using ‘CaspACE, FITC-VAD-fmk In Situ Marker’ (Promega) with or without PI staining using a protocol adapted from [42] (3). *S. cerevisiae* W303-1A cells were exposed to 30 µM C2-phytoceramide or 0.1% (v/v) DMSO for 60 minutes, single stained with FITC or with PI, and double stained with FITC and PI. Most of the cells displaying FITC staining also exhibited compromised membrane integrity (PI staining). **B**. Survival of the metacaspase mutant Δ*yca1* cells after exposure to 30 µM C2-phytoceramide or equivalent volume of solvent (0.1% v/v, DMSO) for 120 min.
(PDF)

Figure S7 **C2-phytoceramide does not trigger autophagy.** **A**. Western blot of whole cell extracts from W303-1A cells expressing *GFP-ATG8*, before (time 0) and after exposure to 30 µM C2-phytoceramide (C2-Phy), or equivalent volume of solvent (DMSO) for 120 min. Positive control (C+) represents cells grown on nitrogen starvation medium for 24 h. The GFP-Atg8 fusion was detected using an anti-GFP antibody (lower panel). The amount of Pgk1 protein was used as a loading control, and detected with an anti-PGK1 antibody (upper panel). **B**. Survival of *S. cerevisiae* W303-1A and *atg5*Δ mutant cells exposed to 30 µM C2-phytoceramide for 120 min. 100% corresponds to the number of CFU at time zero. Values are means ± SE of 3 independent experiments.
(PDF)

Figure S8 **C2-phytoceramide doesn’t interfere with actin polarization in** ***S. cerevisiae***. W303-1A cells were grown in SC galactose to mid-exponential-phase, exposed to 0.1% (v/v) DMSO and 30 µM of C2-phytoceramide, fixed for 15-30 min in 300 µl of formaldehyde 3.7%, and permeabilized with 0.1% Triton X-100 in PBS. After washing 2x with 300 µl of PBS, cells were stained with 1 µl (3 units, from 200 units/ml stock solution) phalloidine rhodamine for 20 min.
(PDF)

Figure S9 **Phosphorylation of Hog1p and effect of pre-incubation under osmotic stress in C2-phytoceramide-induced loss of CFU.** Cells of the W303-1A wild-type (wt) strain were grown in SC galactose to mid-exponential-phase (OD_600 =_0.5-0.6), diluted to OD_600_= 0.2 and then exposed to 30 µM of C2-phytoceramide, or equivalent volume of solvent (0.1% v/v, DMSO) for up to 30 min. At the indicated times, cells were harvested and processed, and the crude protein extracts were analyzed by Western blot. Wt cells grown in YPD medium at 30 °C, non-treated (YPD) or treated with 1 M NaCl-stressed cells (NaCl) for 5 min. were used as control. Total protein extracts were analyzed by SDS-PAGE and blotting with anti-phospho-p38 antibody, which cross-reacts with the dual phosphorylated form of Hog1p (P-Hog1p). The lower membranes were blotted with anti-Hog1p (Hog1p) and with anti-PGK1 (Pgk1p) antibodies as loading controls. **B**. Quantification of P-Hog1p level over time for the experiments described in (A). Cytosolic phosphoglycerate kinase (Pgk1p) level was used to normalize protein amount loaded on the gel. Values are means ± SD of three independent experiments. **C**. Percentage of P.I. positive cells, of cells grown to mid-exponential-phase, exposed 10 min to sorbitol 1M (for Hog1p activation), followed by incubation with 30 µM of C2-phytoceramide and 0.1% (v/v) DMSO for up to 120 min.
(PDF)
