# Supplementary figures and images for: C2-Phytoceramide Perturbs Lipid Rafts and Cell Integrity in Saccharomyces cerevisiae in a Sterol-Dependent Manner
Source: PLoS One. 2013 Sep 11;8(9):e74240. doi: 10.1371/journal.pone.0074240 (PMC3770674; doi:10.1371/journal.pone.0074240)

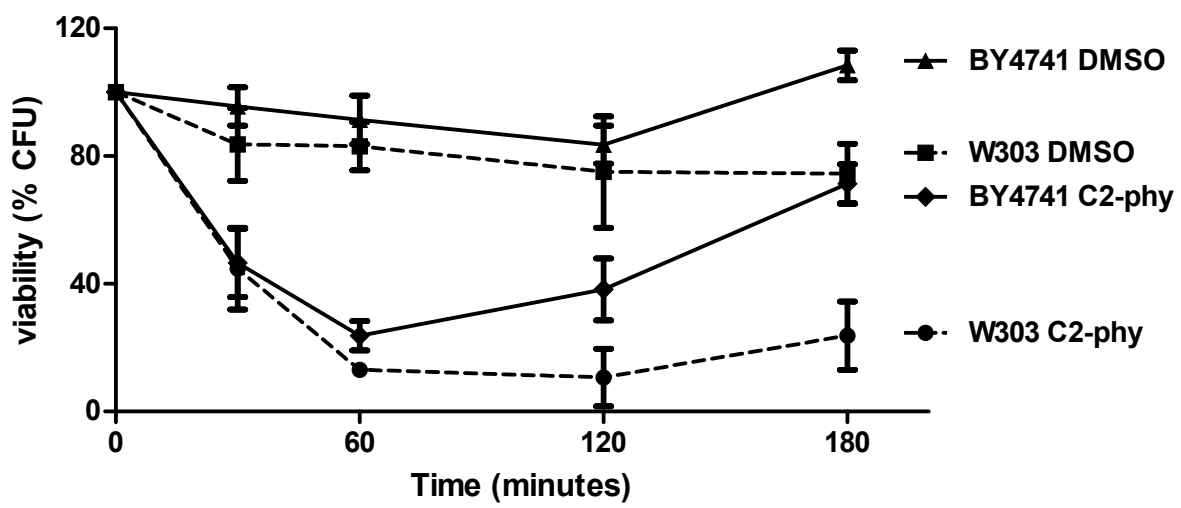

Supplement: Figure S1 — S. cerevisiae W303-1A and BY4741 cells are sensitive to C2-phytoceramide. Survival of W303-1A (full lines) and BY4741 (dashed lines) cells exposed to 30 µM C2-phytoceramide (●,♦) or equivalent volume of solvent (■, ▲). CFU values of C2-treated cells significantly different from DMSO-treated cells, P<0.001, Two-Way ANOVA. All CFU values represent mean ± SE of at least 3 independent experiments, with 5 replicas in each experiment. (PDF) [file pone.0074240.s001.pdf]

A

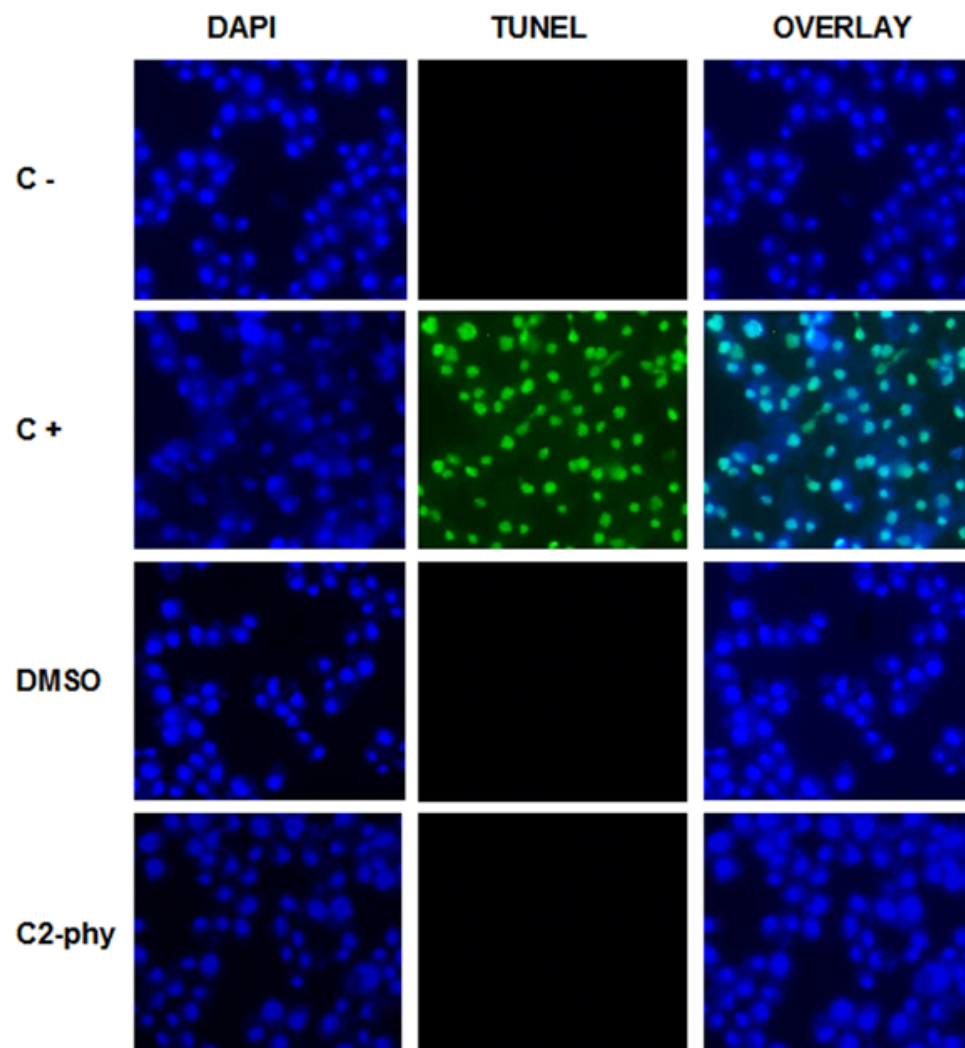

B

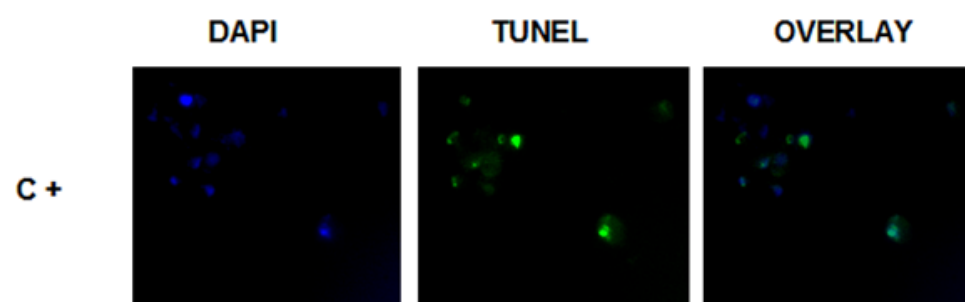

Supplement: Figure S2 — C2-phytoceramide does not induce DNA fragmentation assessed by TUNEL. A. Fluorescence images of TUNEL staining of S. cerevisiae W303-1A treated with 30 µM of C2-phytoceramide or 0.1% (v/v) DMSO for 120 min. Non-treated cells were used as a negative control and DNase I treated cell were used as a positive control for DNA breaks. B. Fluorescence images of TUNEL staining of S. cerevisiae W303-1A treated with 180 mM of acetic acid for 150 min, used as a positive control for induction of DNA breaks during an apoptotic process [41]. The occurrence of DNA strand breaks was determined using the In Situ Cell Death Detection Kit, Fluorescein (Roche Applied Science, Indianapolis, IN) as previously described [18]. (PDF) [file pone.0074240.s002.pdf]

**A**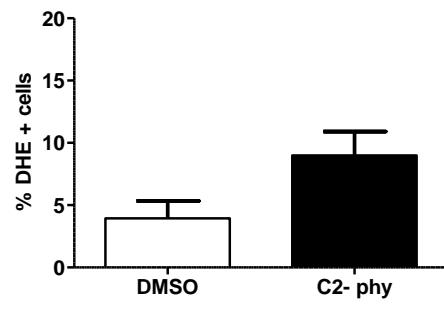**B**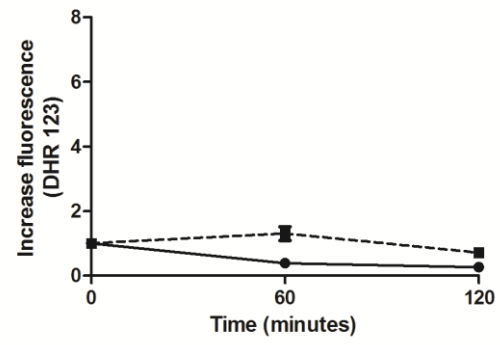**C**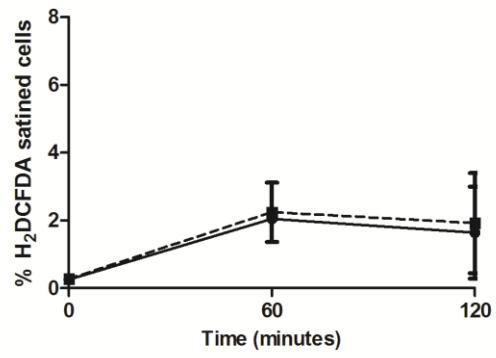**D**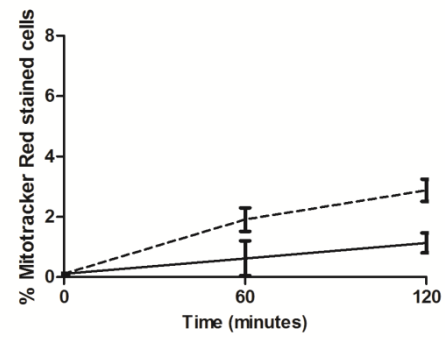

Supplement: Figure S3 — C2-phytoceramide does not lead to significant ROS accumulation. ROS production in S. cerevisiae W303-1A cells exposed to 30 µM C2-phytoceramide or equivalent volume of solvent (0.1% v/v, DMSO) for up 120 min was measured by flow cytometry using: A. dihydroethidium (DHE): Differences between C2-phytoceramide and DMSO treated cells are not significant, P > 0.05 (ns) One-Way ANOVA. Data are given as mean ± SE of at least 3 independent experiments, with 5 replicas in each experiment; B. dihydrorhodamine 123 (DHR 123): For T0, P > 0.05 (ns), T60, P<0.001 and for T120, P<0.01. Two-Way ANOVA. Data are given as mean ± SE of 3 independent experiments; C. 2′, 7′-Dichlorofluorescein diacetate (H 2DCFDA): Differences between C2-phytoceramide and DMSO treated cells are not significant, P > 0.05 (ns) Two-Way ANOVA; D. Mitotracker Red CM-H2XRos :. For T0 and T60, P>0.05 (ns), and for T120, P<0.05. Two-Way ANOVA. Data are given as mean ± SE of 3 independent experiments. Intracellular generation of superoxide anion was monitored using DHE, (Molecular Probes, Eugene, U.S.A.). 1×10^6^ cells were harvested by centrifugation, resuspended in PBS, and stained with 5 µg/ml of dihydroethidium at 30 °C for 30 minutes, in the dark. Fluorescence was measured by flow cytometry. For detection of intracellular ROS with dihydrorhodamine 123 (DHR123) (Molecular Probes, Eugene, OR, USA) a 2.5 mg/ml stock solution in DMSO was added to 106 cells/ml suspended in PBS, to a final concentration of 15 µg/ml. Cells were incubated at 30°C for 90 minutes, in the dark. Results are expressed as ratio values estimated by dividing the mean fluorescence intensity of each sample by the mean fluorescence intensity of time zero. For detection of ROS with H 2DCFDA (Molecular probes), a double staining protocol with PI was used. Conversion of H 2DCFDA to DCF was analyzed in PI negative cells. 106 cells were incubated in culture medium containing 40 µg/ml H2DCFDA at 30 °C for 45 min, in the dark. 2 µg/milliliter of PI was [file pone.0074240.s003.pdf]

**A**

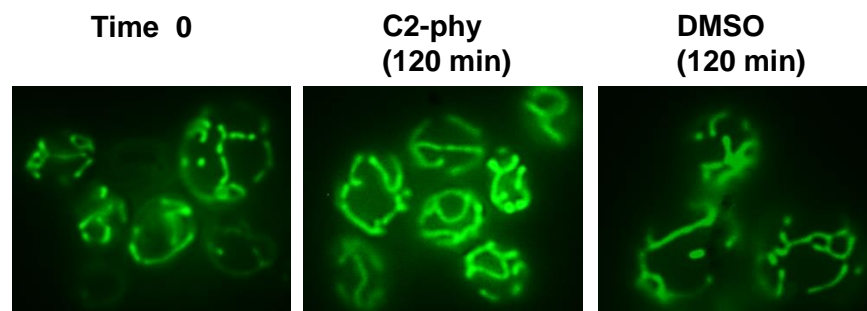

**B**

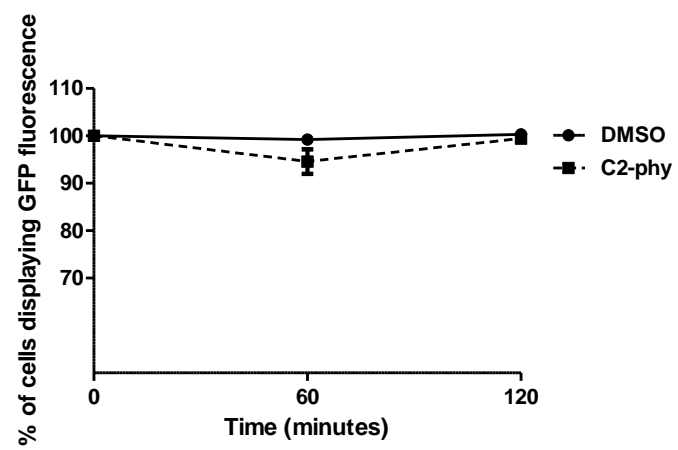

Supplement: Figure S4 — C2-phytoceramide does not induce mitochondrial fragmentation and degradation. A. Mitochondrial morphology of S. cerevisiae W303-1A cells expressing mitochondrial targeted GFP (W303-1A transformed with pYES2-mtGFP), treated with 30 µM C2-phytoceramide or with 0.1% (v/v) DMSO for 120 min. Non-treated cells (time 0) were used as a control. B. Quantification of the percentage of cells displaying GFP fluorescence over the treatment described in (A). Loss of GFP fluorescence was used as a measure of mitochondrial degradation. Values are mean ± SE of 3 independent experiments. (PDF) [file pone.0074240.s004.pdf]

A

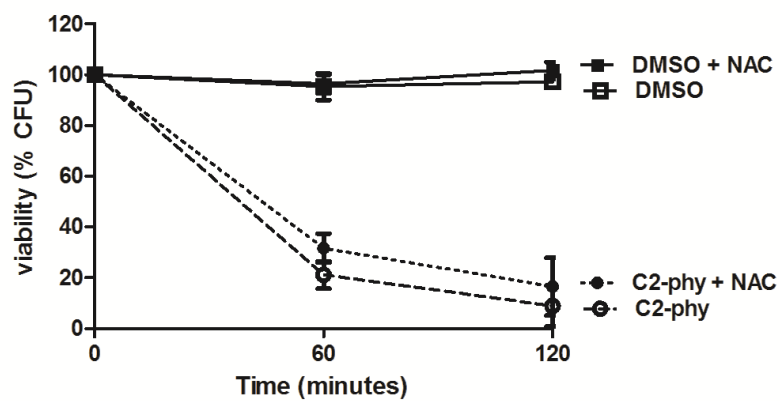

B

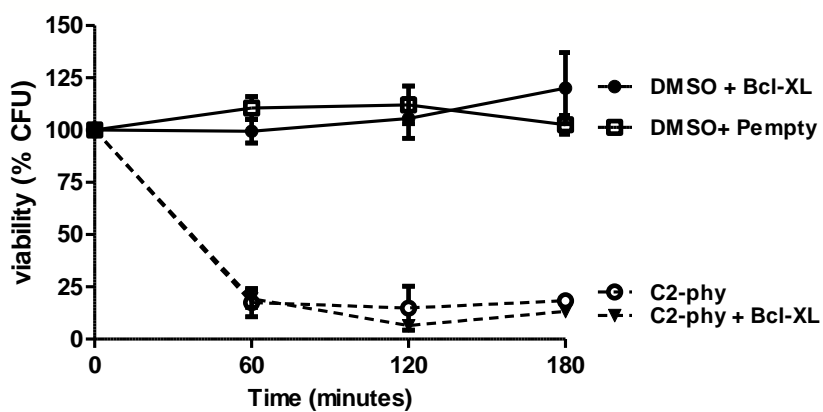

C

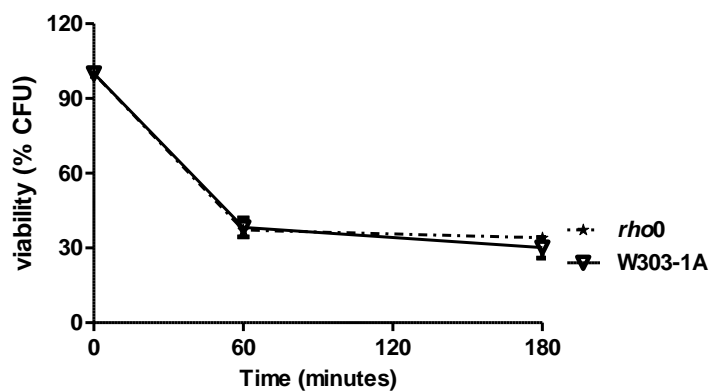

Supplement: Figure S5 — Loss of cell viability induced by C2-phytoceramide could not be inhibited by 20 µM of N-acetylcysteine (NAC), by overexpression of the anti-apoptotic protein Bcl-xL or in a rho0 mutant. A. Survival of S. cerevisiae W303-1A cells exposed to 30 µM C2-phytoceramide (○), 30 µM C2-phytoceramide and 20 µM NAC (●), 0.1% (v/v) DMSO (□) and 0.1% (v/v) DMSO with 20 µM NAC (■) for up to 120 min. B. Survival of S. cerevisiae W303-1A cells overexpressing Bcl-xL, exposed to 30 µM C2-phytoceramide (▼) or 0.1% (v/v) DMSO (●) for up to 180 min. W303-1A cells harboring the empty plasmid (pYES2) treated with 30 µM C2-phytoceramide (○) or 0.1% (v/v) DMSO (□). C. Survival of S. cerevisiae W303-1A cells (∇) and rho0 mutant (*) exposed to 30 µM C2-phytoceramide for up to 120 min. In all experiments 100% corresponds to the number of CFU at time zero. Values are means ± SE of 3 independent experiments. (PDF) [file pone.0074240.s005.pdf]

**A**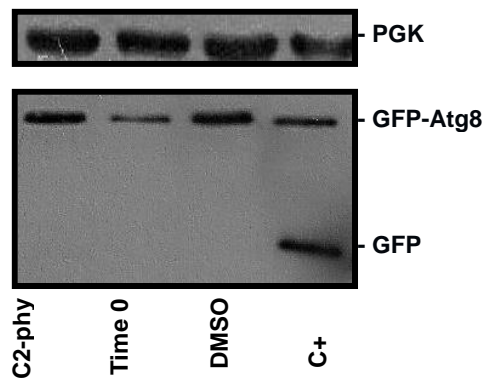**B**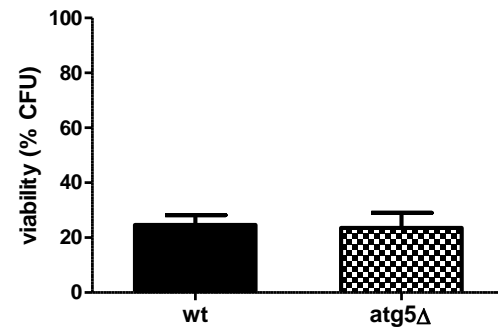

Supplement: Figure S7 — C2-phytoceramide does not trigger autophagy. A. Western blot of whole cell extracts from W303-1A cells expressing GFP-ATG8, before (time 0) and after exposure to 30 µM C2-phytoceramide (C2-Phy), or equivalent volume of solvent (DMSO) for 120 min. Positive control (C+) represents cells grown on nitrogen starvation medium for 24 h. The GFP-Atg8 fusion was detected using an anti-GFP antibody (lower panel). The amount of Pgk1 protein was used as a loading control, and detected with an anti-PGK1 antibody (upper panel). B. Survival of S. cerevisiae W303-1A and atg5Δ mutant cells exposed to 30 µM C2-phytoceramide for 120 min. 100% corresponds to the number of CFU at time zero. Values are means ± SE of 3 independent experiments. (PDF) [file pone.0074240.s007.pdf]

**C2-phy**

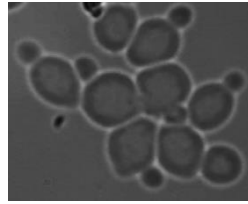

**DMSO**

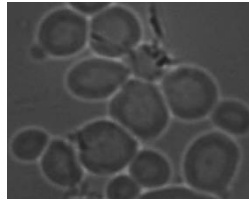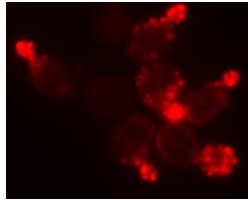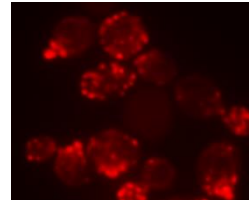

Supplement: Figure S8 — C2-phytoceramide doesn’t interfere with actin polarization in S. cerevisiae. W303-1A cells were grown in SC galactose to mid-exponential-phase, exposed to 0.1% (v/v) DMSO and 30 µM of C2-phytoceramide, fixed for 15-30 min in 300 µl of formaldehyde 3.7%, and permeabilized with 0.1% Triton X-100 in PBS. After washing 2x with 300 µl of PBS, cells were stained with 1 µl (3 units, from 200 units/ml stock solution) phalloidine rhodamine for 20 min. (PDF) [file pone.0074240.s008.pdf]

**A**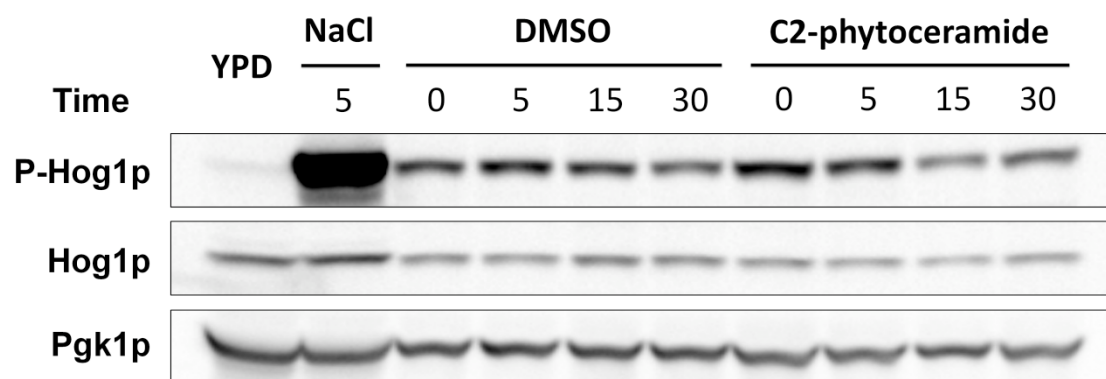**B**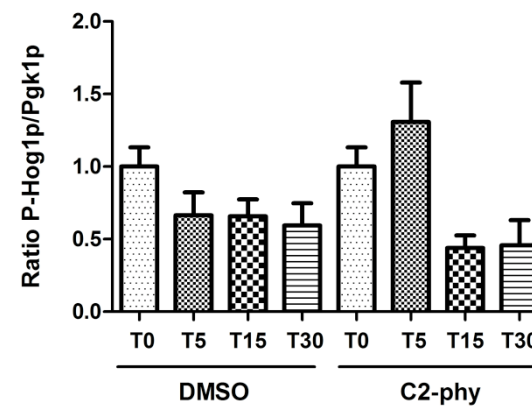**C**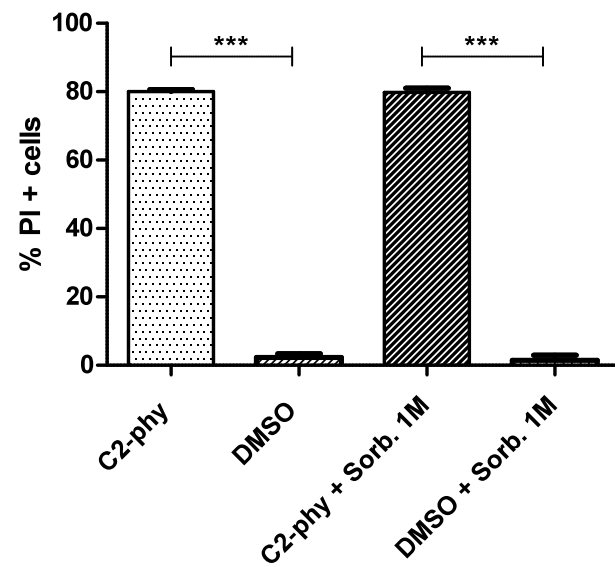

Supplement: Figure S9 — Phosphorylation of Hog1p and effect of pre-incubation under osmotic stress in C2-phytoceramide-induced loss of CFU. Cells of the W303-1A wild-type (wt) strain were grown in SC galactose to mid-exponential-phase (OD600 =0.5-0.6), diluted to OD600= 0.2 and then exposed to 30 µM of C2-phytoceramide, or equivalent volume of solvent (0.1% v/v, DMSO) for up to 30 min. At the indicated times, cells were harvested and processed, and the crude protein extracts were analyzed by Western blot. Wt cells grown in YPD medium at 30 °C, non-treated (YPD) or treated with 1 M NaCl-stressed cells (NaCl) for 5 min. were used as control. Total protein extracts were analyzed by SDS-PAGE and blotting with anti-phospho-p38 antibody, which cross-reacts with the dual phosphorylated form of Hog1p (P-Hog1p). The lower membranes were blotted with anti-Hog1p (Hog1p) and with anti-PGK1 (Pgk1p) antibodies as loading controls. B. Quantification of P-Hog1p level over time for the experiments described in (A). Cytosolic phosphoglycerate kinase (Pgk1p) level was used to normalize protein amount loaded on the gel. Values are means ± SD of three independent experiments. C. Percentage of P.I. positive cells, of cells grown to mid-exponential-phase, exposed 10 min to sorbitol 1M (for Hog1p activation), followed by incubation with 30 µM of C2-phytoceramide and 0.1% (v/v) DMSO for up to 120 min. (PDF) [file pone.0074240.s009.pdf]
