# Supplementary material for: C2-Phytoceramide Perturbs Lipid Rafts and Cell Integrity in Saccharomyces cerevisiae in a Sterol-Dependent Manner
Source: PLoS One. 2013 Sep 11;8(9):e74240. doi: 10.1371/journal.pone.0074240 (PMC3770674; doi:10.1371/journal.pone.0074240)

A

Time (min.)	Single stainning FITC (%)		Single stainning PI (%)		Double stainning FITC/PI (%)	
	DMSO	C2-PHY	DMSO	C2-PHY	DMSO	C2-PHY
0	1.44	1.44	0.13	0.13	0.67	0.67
60	0.63	2.13	1.17	3.44	0.70	3.92

B

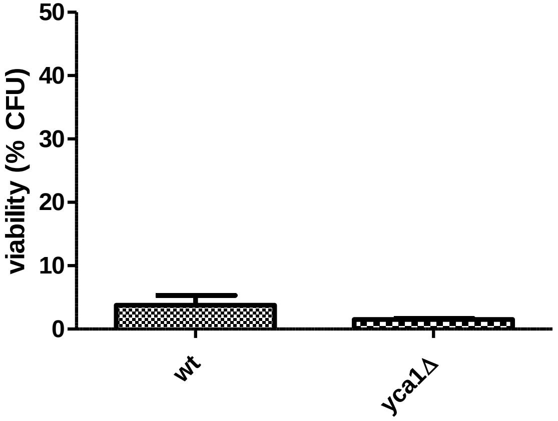

Supplement: Figure S6 — Caspases activation and Yca1p are not involved in C2-phytoceramide-induced loss of CFU. A. Caspase activation assessed by flow cytometry using ‘CaspACE, FITC-VAD-fmk In Situ Marker’ (Promega) with or without PI staining using a protocol adapted from [42] (3). S. cerevisiae W303-1A cells were exposed to 30 µM C2-phytoceramide or 0.1% (v/v) DMSO for 60 minutes, single stained with FITC or with PI, and double stained with FITC and PI. Most of the cells displaying FITC staining also exhibited compromised membrane integrity (PI staining). B. Survival of the metacaspase mutant Δyca1 cells after exposure to 30 µM C2-phytoceramide or equivalent volume of solvent (0.1% v/v, DMSO) for 120 min. (PDF) [file pone.0074240.s006.pdf]
